# Coffee consumption and skeletal muscle mass: WASEDA’S Health Study

**DOI:** 10.1017/S0007114522003099

**Published:** 2023-07-14

**Authors:** Ryoko Kawakami, Kumpei Tanisawa, Tomoko Ito, Chiyoko Usui, Kaori Ishii, Isao Muraoka, Katsuhiko Suzuki, Shizuo Sakamoto, Mitsuru Higuchi, Koichiro Oka

**Affiliations:** 1 Faculty of Sport Sciences, Waseda University, 2-579-15 Mikajima, Tokorozawa, Saitama 359-1192, Japan; 2 Waseda Institute for Sport Sciences, Waseda University, 2-579-15 Mikajima, Tokorozawa, Saitama 359-1192, Japan; 3 Department of Food and Nutrition, Tokyo Kasei University, 1-18-1 Kaga, Itabashi, Tokyo 173-8602, Japan; 4 Faculty of Sport Science, Surugadai University, 698 Azu, Hanno, Saitama 357-8555, Japan

**Keywords:** Beverages, Body composition, Caffeine, Sarcopenia

## Abstract

Few studies have examined the association between coffee consumption and muscle mass; their results are conflicting. Therefore, we examined the association between coffee consumption and low muscle mass prevalence. We also performed an exploratory investigation of the potential effect modification by demographic, health status-related and physical activity-related covariates. This cross-sectional study included 2085 adults aged 40–87 years. The frequency of coffee consumption was assessed using a self-administered questionnaire. Muscle mass was assessed as appendicular skeletal muscle mass/height^2^ using a multifrequency bioelectrical impedance analyser. We defined low muscle mass using cut-offs recommended by the Asian Working Group for Sarcopenia. Multivariable-adjusted OR for low muscle mass prevalence were estimated using a logistic regression model. The prevalence of low muscle mass was 5·4 % (*n* 113). Compared with the lowest coffee consumption group (< 1 cup/week), the multivariable-adjusted OR (95 % CI) of low muscle mass prevalence were 0·62 (0·30, 1·29) for 1–3 cups/week, 0·53 (0·29, 0·96) for 4–6 cups/week or 1 cup/d and 0·28 (0·15, 0·53) for ≥ 2 cups/d (*P* for trend < 0·001). There were no significant interactions among the various covariates after Bonferroni correction. In conclusion, coffee consumption may be inversely associated with low muscle mass prevalence.

Sarcopenia is defined as a progressive and generalised skeletal muscle disorder involving an accelerated loss of muscle mass and function^(1)^. Sarcopenia is associated with a wide range of adverse health-related outcomes, such as metabolic diseases, falls, fractures and all-cause mortality^(2,3)^. Therefore, it is of public health significance to identify modifiable lifestyle risk factors that can lower the risk of sarcopenia.

Coffee is one of the most frequently consumed beverages worldwide. Moderate coffee consumption has been reported to be associated with lower risks of specific cancers, CVD, metabolic diseases and all-cause mortality^(4,5)^. Animal studies have suggested that coffee has a beneficial effect on skeletal muscle mass improvement^(6–8)^. Although the molecular mechanisms underlying the relationships between coffee consumption and muscle mass have not been fully elucidated, anti-inflammatory and anti-oxidative effects, autophagy, downregulation of myostatin and upregulation of insulin-like growth factor may be involved in the effect of coffee on increased muscle mass^(6–8)^. Ergogenic effects of caffeine on exercise performance, including muscle endurance, muscle strength, anaerobic power and aerobic endurance, have been widely studied^(9)^. However, to the best of our knowledge, studies on the association between coffee consumption and muscle mass are limited, with only three cross-sectional studies and one longitudinal study^(10–13)^, and these have shown conflicting results. Two cross-sectional studies, one on middle-aged and older Japanese men and women^(10)^ and the other on older Korean men^(11)^, have reported that higher coffee consumption was associated with higher muscle mass. However, a cross-sectional study on Korean men and women aged 40 years and older reported that light coffee consumption was associated with a lower prevalence of low muscle mass in men but not in women^(12)^. Furthermore, heavy coffee consumption was associated with a higher prevalence of obesity in women^(12)^; however, there might have been heterogeneity regarding obesity status in the relationship between coffee consumption and muscle mass. A longitudinal study reported that there was no clear association between changes in coffee consumption and concurrent changes in the fat-free mass index^(13)^. One reason for the lack of agreement in these observational studies may be the effect modification, whereby certain factors influence the relationship between coffee and muscle mass. However, the potential effect modification has not been examined in previous epidemiologic studies.

This cross-sectional study aimed to further assess the association between coffee consumption and the prevalence of low muscle mass in middle-aged and older adults. Additionally, we performed an exploratory investigation of effect modification in the association by demographic, health status-related and physical activity-related covariates.

## Methods

### Participants

This was a cross-sectional study performed using baseline survey data from a prospective cohort study based at the Waseda University in Japan^(14–17)^. The Waseda Alumni’s Sports, Exercise, Daily Activity, Sedentariness and Health Study (WASEDA’S Health Study) included Waseda University’s alumni and their spouses aged 40 years or older. The participants voluntarily selected one of four courses (cohorts A–D) with different measurement items^(16)^. As cohorts A and B completed non-face-to-face surveys using the internet and/or postal mail, muscle mass was assessed by a bioelectrical impedance analyser (BIA) only in cohorts C and D. The cohort in our study comprised 2538 middle-aged and older men and women who underwent muscle mass measurements in cohorts C or D between March 2015 and March 2020.

Potential participants were excluded for the following reasons: (1) incomplete results on the internet-based questionnaire (*n* 81); (2) incomplete dietary survey data (*n* 20); (3) extreme levels of self-reported energy intake (< 600 kcal/d [< 2510.4 kJ/d] or > 4000 kcal/d [> 16736.0 kJ/d]; *n* 10); (4) extreme levels of self-reported physical activity (*n* 26); (5) pregnant or lactating status (*n* 14); and (6) self-reported history of cancer (*n* 146), stroke (*n* 28), heart diseases (*n* 41), kidney failure (*n* 5), liver cirrhosis and hepatitis (*n* 9) and diabetes mellitus (*n* 73). Thus, the analysis included 2085 participants (1296 men and 789 women). A flow diagram of the participant enrolment is presented in online Supplementary Fig. 1.

### Ethical approval

All participants received an explanation of the research prior to the measurements and provided written informed consent. The study was approved by the Research Ethics Committee of Waseda University (approval numbers: 2014–095, 2014-G002, 2018–320 and 2018-G001) and was conducted in accordance with the Declaration of Helsinki.

### Assessment of coffee consumption and nutritional intake

Energy intake (kcal/d) and protein intake (g/d) were assessed using the brief-type self-administered diet history questionnaire (BDHQ), which has been validated in Japanese adults^(18,19)^. Consumption frequencies of coffee (< 1 cup/week, 1–3 cups/week, 4–6 cups/week or 1 cup/d and ≥ 2 cups/d), green tea (< 1 cup/week, 1–3 cups/week, 4–6 cups/week or 1 cup/d and ≥ 2 cups/d) and alcohol (< 1 d/week, 1–3 d/week, 4–6 d/week and every day) were each assessed using a single item included in the BDHQ. Another category of coffee consumption frequency (none, < 1 cup/week, 1 cup/week, 2–3 cups/week, 4–6 cups/week, 1 cup/d, 2–3 cups/d and ≥ 4 cups/d) was also assessed, and a sub-analysis was performed.

### Assessment of muscle mass

Anthropometric measurements were performed in the morning by trained researchers after the participants had fasted for a minimum of 12 h. Height and body composition were measured with the participants wearing light clothing and no shoes. Appendicular skeletal muscle mass (ASM) was estimated using a multifrequency BIA (MC-980A, Tanita Corp.), as described previously^(14)^. To adjust for the effects of an individual’s physique, we divided the ASM (kg) by the square of height (m). Our previous study confirmed a strong correlation between ASM/height^2^ measured with the same BIA and ASM/height^2^ measured using dual-energy X-ray absorptiometry (Horizon A, Hologic Inc.) (*r* = 0·88 for men, *r* = 0·84 for women)^(14)^. The inter-instrument reliability between the two measurement methods for ASM was good (intraclass correlation coefficient = 0·88 for men and 0·76 for women)^(15)^. We defined low muscle mass based on the cut-offs for BIA-measured muscle mass values, as recommended by the Asian Working Group for Sarcopenia 2019^(20)^; the ASM/height^2^ cut-offs were 7·0 and 5·7 kg/m^2^ for men and women, respectively.

### Assessment of other variables

Leisure-time physical activity was assessed using the Global Physical Activity Questionnaire developed by the World Health Organisation^(21)^. Following the Global Physical Activity Questionnaire analysis guide, moderate-intensity and vigorous-intensity activities were assigned four and eight metabolic equivalents of task (MET), respectively. The amount of leisure-time physical activity (MET-min/week) was calculated by summing the amount of moderate- and vigorous-intensity leisure-time physical activities. Marital status (married and unmarried), education level (junior high or high school, junior college or technical college and university or higher), annual household income (< 3 000 000 JPY, 3 000 000–4 999 999 JPY, 5 000 000–6 999 999 JPY, 7 000 000–9 999 999 JPY and ≥ 10 000 000 JPY), cigarette smoking status (current smoker, former smoker and never smoked) and antihypertensive and anti-dyslipidaemic drug usage (user and non-user) were assessed using internet-based questionnaires.

### Statistical analysis

For descriptive data, continuous variables are shown as median values (interquartile ranges), while categorical variables are presented as numbers (%). To investigate the association between coffee consumption and low muscle mass prevalence, we calculated multivariable-adjusted OR and 95 % CI using a logistic regression model with low muscle mass prevalence as the dependent variable and coffee consumption with the covariates as the independent variable. First, we calculated the OR, adjusting only for age (years) and sex (men and women). We subsequently expanded the models with the following covariates: cohorts (cohort C and cohort D), body fat (%), marital status (married and unmarried), education level (junior high or high school, junior college or technical college and university or higher), annual household income (< 3 000 000 JPY, 3 000 000–4 999 999 JPY, 5 000 000–6 999 999 JPY, 7 000 000–9 999 999 JPY and ≥ 10 000 000 JPY), cigarette smoking status (current smoker, former smoker and never smoked), frequency of alcohol consumption (< 1 d/week, 1–3 d/week, 4–6 d/week and every day), antihypertensive drug usage (user and non-user), anti-dyslipidaemic drug usage (user and non-user), energy intake (kcal/d), protein intake (g/d), green tea consumption (< 1 cup/week, 1–3 cups/week, 4–6 cups/week or 1 cup/d and ≥ 2 cups/d) and leisure-time physical activity (MET-min/week). These covariates were selected based on previous studies examining the association between coffee consumption and muscle mass^(10,11)^. We calculated the E-values for the model covariates. The E-value represents the minimum strength of an association that an uncontrolled covariate would need to have with both the exposure (coffee consumption) and the outcome (low muscle mass) to have a significant impact on the OR^(22,23)^.

Obesity was defined as a body fat percentage of ≥ 25 % for men and ≥ 30 % for women^(24)^. Participants were classified into two groups by sex based on the median energy intake, protein intake or leisure-time physical activity. To investigate the interactions of the dichotomised covariates and three levels of coffee consumption with low muscle mass prevalence, the interaction terms (i.e., dichotomised covariates × three levels of coffee consumption) were entered together into the multivariate logistic regression model. These factors included the following: age (< 60 *v*. ≥ 60 years), sex (men *v*. women), body fat (< 25 *v*. ≥ 25 % for men, < 30 *v*. ≥ 30 % for women), marital status (married *v*. unmarried), education level (higher than university graduation *v*. lower education), annual household income (≥ 10 000 000 JPY *v*. lower income), cigarette smoking status (never smoked *v*. has smoked), alcohol consumption (< 1 *v*. ≥ 1 d/week), antihypertensive drug usage (user *v*. non-user), anti-dyslipidaemic drug usage (user *v*. non-user), energy intake (< 1990·0 *v*. ≥ 1990·0 kcal/d for men, < 1677·6 *v*. ≥ 1677·6 kcal/d for women), protein intake (< 74·6 *v*. ≥ 74·6 g/d for men, < 67·7 *v*. ≥ 67·7 g/d for women), green tea consumption (< 4 *v*. ≥ 4 cups/week) and leisure-time physical activity (< 480 *v*. ≥ 480 MET-min/week for men, < 240 *v*. ≥ 240 MET-min/week for women). Furthermore, we performed subgroup analyses for dichotomised values of age, sex, body fat, antihypertensive drug usage and leisure-time physical activity.

All statistical analyses were performed using SPSS Statistics version 27 (IBM Corp) and Stata version 17 (StataCorp). Statistical significance was defined by a *P*-value of < 0·05 for the two-sided tests. In interaction analysis, Bonferroni correction for multiple testing was applied to the significance level (14 tests, *P* for interaction < 0·003).

## Results

Table 1 shows the characteristics of the participants according to the coffee consumption categories. The median age of the participants was 52 years (interquartile range, 46–59 years). The prevalence rate of low muscle mass was 5·4 % (*n* 113) and the median ASM/height^2^ was 7·6 kg/m^2^ (interquartile range, 6·5–8·3 kg/m^2^). In terms of coffee consumption, 10·0 % (*n* 208) consumed < 1 cup/week, 10·9 % (*n* 228) consumed 1–3 cups/week, 32·6 % (*n* 679) consumed 4–6 cups/week or 1 cup/d and 46·5 % (*n* 970) consumed ≥ 2 cups/d of coffee. The group with the highest coffee consumption (≥ 2 cups/d) tended to have a higher energy and protein intake and lower green tea consumption, and there were more current smokers and fewer antihypertensive drug users in this group.

**Table 1.** Characteristics of the participants according to coffee consumption

			Coffee consumption							
	Total (*n* 2085)		< 1 cup/week (*n* 208)		1–3 cups/week (*n* 228)		4–6 cups/week or 1 cup/d (*n* 679)		≥ 2 cups/d (*n* 970)	
	Median	IQR	Median	IQR	Median	IQR	Median	IQR	Median	IQR
Men (%)	62·2		63·9		61·0		61·3		62·7	
Cohort C (%)	45·7		49·5		46·5		47·1		43·7	
Age (years)	52	46–59	51	45–61	50	46–60	52	47–61	52	46–58
Height (cm)	166·2	160·2–172·3	165·4	160·7–171·7	166·4	160·8–173·0	165·8	160·1–171·9	166·7	160·0–172·4
Body weight (kg)	62·5	54·4–70·8	62·3	54·2–69·3	62·4	54·4–71·7	62·7	54·2–70·4	62·3	54·4–70·9
Body fat (%)	22·8	18·6–27·6	23·3	18·7–28·1	24·0	19·6–28·5	22·6	18·7–27·5	22·5	18·2–27·4
ASM/height^2^ (kg/m^2^)	7·6	6·5–8·3	7·6	6·4–8·2	7·7	6·5–8·4	7·5	6·5–8·3	7·6	6·5–8·4
Low muscle mass (%)*	5·4		10·1		6·6		6·3		3·5	
Leisure-time physical activity (MET-min/week)	360	0–960	240	0–840	360	0–960	320	0–960	480	0–960
Energy intake (kcal/d)	1867	1559–2255	1767	1456–2160	1780	1500–2149	1850	1575–2261	1910	1589–2290
Protein intake (g/d)	72	59–89	68	57–86	69	55–88	72	59–89	73	60–92
Green tea consumption (%)										
< 1 cup/week	16·4		20·7		13·6		13·0		18·5	
1–3 cups/week	20·9		18·3		18·0		20·6		22·3	
4–6 cups/week or 1 cup/d	27·6		15·4		26·8		30·6		28·4	
≥ 2 cups/d	35·2		45·7		41·7		35·8		30·9	
Antihypertensive drug usage (%)	13·0		17·3		14·9		14·6		10·6	
Anti-dyslipidaemic drug usage (%)	8·7		10·1		10·5		9·0		7·7	
Married (%)	84·5		83·2		81·1		86·2		84·3	
Education level (%)										
Junior high or high school	2·2		2·4		2·2		2·5		2·0	
Junior college or technical college	6·8		5·8		7·5		7·2		6·6	
University or higher	91·0		91·8		90·4		90·3		91·4	
Household income (%)										
< 3 000 000 JPY	6·7		9·1		5·7		6·9		6·3	
3 000 000–4 999 999 JPY	14·0		15·9		16·2		15·8		11·8	
5 000 000–6 999 999 JPY	15·9		14·4		14·9		15·2		17·0	
7 000 000–9 999 999 JPY	22·4		22·1		25·9		22·8		21·3	
≥ 10 000 000 JPY	41·0		38·5		37·3		39·3		43·6	
Cigarette smoking status (%)										
Current smoker	6·8		5·3		3·9		5·6		8·6	
Former smoker	31·0		26·0		34·6		32·4		30·3	
Never smoked	62·2		68·8		61·4		62·0		61·1	
Alcohol consumption (%)										
< 1 d/week	34·5		42·3		34·2		33·3		33·8	
1–3 d/week	25·0		25·0		25·9		22·8		26·4	
4–6 d/week	19·6		13·9		18·9		22·5		19·0	
Every day	20·8		18·8		21·1		21·4		20·8	

ASM, appendicular skeletal muscle mass; IQR, interquartile range; MET, metabolic equivalent of task.

Data are expressed as medians and IQR or %.

* Low muscle mass was defined based on the definition by the Asian Working Group for Sarcopenia 2019. The recommended cut-offs for appendicular skeletal muscle mass/height^2^ were determined by bioelectrical impedance analysis were < 7·0 kg/m^2^ and < 5·7 kg/m^2^ for men and women, respectively.

Table 2 shows the adjusted OR for low muscle mass prevalence for each covariate. Men, younger age, higher body fat and higher leisure-time physical activity were associated with a lower prevalence of low muscle mass.

**Table 2.** OR for the prevalence of low muscle mass according to the study covariates

	No. of participants	No. of cases	No. of cases per 1000 participants	OR	95 % CI	*P* for trend
Sex						
Women	789	61	77	1·00	Reference	
Men	1296	52	40	0·17	0·10, 0·31	–
Cohort						
Cohort C	953	58	61	1·00	Reference	
Cohort D	1132	55	49	0·89	0·59, 1·34	–
Age						
Per 10 years	2085	113	54	2·37	1·82, 3·07	< 0·001
Body fat						
Per 10 %	2085	113	54	0·35	0·24, 0·50	< 0·001
Leisure-time physical activity						
Per 100 MET-min/week	2085	113	54	0·96	0·93, 0·98	0·002
Energy intake						
Per 100 kcal/d	2085	113	54	1·01	0·94, 1·09	0·696
Protein intake						
Per 10 g/d	2085	113	54	0·93	0·80, 1·08	0·345
Green tea consumption						
< 1 cup/week	341	21	62	1·00	Reference	
1–3 cups/week	435	15	34	0·54	0·27, 1·11	
4–6 cups/week or 1 cup/d	576	32	56	0·89	0·48, 1·63	
≥ 2 cups/d	733	45	61	0·82	0·45, 1·48	0·997
Antihypertensive drug usage						
Users	272	10	37	1·00	Reference	
Non-users	1813	103	57	1·76	0·85, 3·65	–
Anti-dyslipidaemic drug usage						
Users	181	6	33	1·00	Reference	
Non-users	1904	107	56	1·71	0·69, 4·22	–
Marital status						
Unmarried	324	15	46	1·00	Reference	
Married	1761	98	56	1·23	0·65, 2·31	–
Education level						
Junior high or high school	46	3	65	1·00	Reference	
Junior college or technical college	142	10	70	1·38	0·34, 5·66	
University or higher	1897	100	53	1·69	0·47, 6·09	0·365
Household income						
< 3 000 000 JPY	140	12	86	1·00	Reference	
3 000 000–4 999 999 JPY	291	17	58	0·63	0·28, 1·45	
5 000 000–6 999 999 JPY	332	24	72	1·13	0·51, 2·47	
7 000 000–9 999 999 JPY	467	21	45	0·80	0·36, 1·79	
≥ 10 000 000 JPY	855	39	46	0·87	0·41, 1·88	0·929
Cigarette smoking status						
Current smoker	141	6	43	1·00	Reference	
Former smoker	647	31	48	0·78	0·30, 1·99	
Never smoked	1297	76	59	0·77	0·30, 1·95	0·712
Alcohol consumption						
< 1 d/week	720	43	60	1·00	Reference	
1–3 d/week	522	21	40	0·97	0·55, 1·74	
4–6 d/week	409	23	56	1·29	0·72, 2·31	
Every day	434	26	60	1·29	0·72, 2·31	0·301

MET, metabolic equivalent of task.

Low muscle mass was defined based on the definition by the Asian Working Group for Sarcopenia 2019. The recommended cut-offs for appendicular skeletal muscle mass/height^2^ were determined by bioelectrical impedance analysis were < 7·0 kg/m^2^ and < 5·7 kg/m^2^ for men and women, respectively.

Adjusted for all items in the table plus coffee consumption (< 1 cup/week, 1–3 cups/week, 4–6 cups/week or 1 cup/d and ≥ 2 cups/d).

Table 3 shows the adjusted OR for low muscle mass prevalence according to coffee consumption. There was an inverse association between coffee consumption and low muscle mass prevalence (*P* for trend < 0·001). Compared with the lowest coffee consumption group (< 1 cup/week), multivariable-adjusted OR (95 % CI) for low muscle mass prevalence were 0·62 (0·30, 1·29), 0·53 (0·29, 0·96) and 0·28 (0·15, 0·53) in the 1–3 cups/week group, 4–6 cups/week or 1 cup/d group and ≥ 2 cups/d group, respectively. The E-value (model 2) of the OR for the ≥ 2 cups/d group was 6·60. We also performed a sub-analysis using another category of coffee consumption frequency in eight groups: none, < 1 cup/week, 1 cup/week, 2–3 cups/week, 4–6 cups/week, 1 cup/d, 2–3 cups/d and ≥ 4 cups/d. The association between coffee consumption and low muscle mass prevalence in the sub-analysis was similar to that found in the main analysis (*P* for trend < 0·001) (online Supplementary Table 1).

**Table 3.** OR for the prevalence of low muscle mass according to coffee consumption

	Coffee consumption								*P* for trend
	< 1 cup/week		1–3 cups/week		4–6 cups/week or 1 cup/d		≥ 2 cups/d		
	OR	95 % CI	OR	95 % CI	OR	95 % CI	OR	95 % CI	
No. of participants	208		228		679		970		
No. of cases	21		15		43		34		
No. of cases per 1000 participants	101		66		63		35		
Model 1 OR	1·00	Reference	0·60	0·29, 1·20	0·56	0·32, 0·98	0·32	0·18, 0·57	< 0·001
Model 2 OR	1·00	Reference	0·62	0·30, 1·29	0·53	0·29, 0·96	0·28	0·15, 0·53	< 0·001

MET, metabolic equivalent of task.

Low muscle mass was defined based on the definition by the Asian Working Group for Sarcopenia 2019. The recommended cut-offs for appendicular skeletal muscle mass/height^2^ were determined by bioelectrical impedance analysis were < 7·0 kg/m^2^ and < 5·7 kg/m^2^ for men and women, respectively.

Model 1: Adjusted for age (years) and sex (men, women).

Model 2: Adjusted for model 1 plus cohort (cohort C, cohort D), body fat (%), marital status (married, unmarried), education level (junior high or high school, junior college or technical college, university or higher), household income (< 3 000 000 JPY, 3 000 000–4 999 999 JPY, 5 000 000–6 999 999 JPY, 7 000 000–9 999 999 JPY, ≥ 10 000 000 JPY), cigarette smoking status (current smoker, former smoker, never smoked), alcohol consumption (< 1 d/week, 1–3 d/week, 4–6 d/week, every day), antihypertensive drug usage (user, non-user), anti-dyslipidaemic drug usage (user, non-user), energy intake (kcal/d), protein intake (g/d), green tea consumption (< 1 cup/week, 1–3 cups/week, 4–6 cups/week or 1 cup/d, ≥ 2 cups/d) and leisure-time physical activity (MET-min/week).

Table 4 shows the adjusted OR for low muscle mass prevalence according to coffee consumption in the subgroup analyses for dichotomised values of age, sex, body fat, antihypertensive drug usage and leisure-time physical activity. There were no significant interactions between coffee consumption and low muscle mass prevalence with the following factors: marital status (*P* for interaction = 0·318), education level (*P* for interaction = 0·170), household income (*P* for interaction = 0·216), cigarette smoking status (*P* for interaction = 0·974), alcohol consumption (*P* for interaction = 0·669), anti-dyslipidaemic drug usage (*P* for interaction = 0·658), energy intake (*P* for interaction = 0·136), protein intake (*P* for interaction = 0·358) and green tea consumption (*P* for interaction = 0·513). The inverse association between coffee consumption and low muscle mass prevalence was similar between men and women (*P* for interaction = 0·533). There were no significant interactions between low muscle mass prevalence and coffee consumption with age (*P* for interaction = 0·903) and body fat (*P* for interaction = 0·119). A tendency for interaction of low muscle mass prevalence with antihypertensive drug usage and coffee consumption was noted, but it was not significant after Bonferroni correction (*P* for interaction = 0·025). An inverse association between coffee consumption and low muscle mass prevalence was noted among non-users of antihypertensive drugs (*P* for trend < 0·001) but not among antihypertensive drug users (*P* for trend = 0·589). A tendency for interaction of leisure-time physical activity and coffee consumption with low muscle mass prevalence was also noted, but it was not significant after Bonferroni correction (*P* for interaction = 0·005). There was an inverse association between coffee consumption and low muscle mass prevalence among adults who performed physical activity (*P* for trend < 0·001), but not among adults with physical inactivity (*P* for trend = 0·419).

**Table 4.** OR for the prevalence of low muscle mass according to coffee consumption in the subgroup analyses by study covariates

	Coffee consumption						*P* for trend
	< 3 cups/week		4–6 cups/week or 1 cup/d		≥ 2 cups/d		
	OR	95 % CI	OR	95 % CI	OR	95 % CI	
Age (*P* for interaction = 0·903)*							
Middle-aged adults (*n* 1569)							
No. of participants	318		492		759		
No. of cases	25		20		25		
No. of cases per 1000 participants	79		41		33		
OR§	1·00	Reference	0·39	0·20, 0·75	0·32	0·17, 0·60	0·001
Older adults (*n* 516)							
No. of participants	118		187		211		
No. of cases	11		23		9		
No. of cases per 1000 participants	93		123		43		
OR§	1·00	Reference	1·45	0·63, 3·36	0·36	0·13, 1·01	0·035
Sex (*P* for interaction = 0·533)							
Men (*n* 1296)							
No. of participants	272		416		608		
No. of cases	16		20		16		
No. of cases per 1000 participants	59		48		26		
OR||	1·00	Reference	0·74	0·36, 1·53	0·42	0·19, 0·89	0·021
Women (*n* 789)							
No. of participants	164		263		362		
No. of cases	20		23		18		
No. of cases per 1000 participants	122		87		50		
OR||	1·00	Reference	0·57	0·28, 1·16	0·32	0·15, 0·67	0·003
Body fat (*P* for interaction = 0·119)†							
Adults without obesity (*n* 1529)							
No. of participants	308		500		721		
No. of cases	34		36		29		
No. of cases per 1000 participants	110		72		40		
OR§	1·00	Reference	0·56	0·33, 0·96	0·31	0·18, 0·54	< 0·001
Adults with obesity (*n* 556)							
No. of participants	128		179		249		
No. of cases	2		7		5		
No. of cases per 1000 participants	16		39		20		
OR§	1·00	Reference	3·12	0·50, 19·57	0·95	0·14, 6·63	0·688
Antihypertensive drug usage (*P* for interaction = 0·025)							
Non-users (*n* 1813)							
No. of participants	366		580		867		
No. of cases	35		39		29		
No. of cases per 1000 participants	96		67		33		
OR¶	1·00	Reference	0·63	0·38, 1·06	0·30	0·17, 0·52	< 0·001
Users (*n* 272)							
No. of participants	70		99		103		
No. of cases	1		4		5		
No. of cases per 1000 participants	14		40		49		
OR¶	1·00	Reference	1·16	0·09, 15·55	1·84	0·15, 22·28	0·589
Leisure-time physical activity (*P* for interaction = 0·005)‡							
Physically inactive adults (*n* 985)							
No. of participants	221		332		432		
No. of cases	14		17		22		
No. of cases per 1000 participants	63		51		51		
OR§	1·00	Reference	0·68	0·30, 1·51	0·70	0·33, 1·50	0·419
Physically active adults (*n* 1100)							
No. of participants	215		347		538		
No. of cases	22		26		12		
No. of cases per 1000 participants	102		75		22		
OR§	1·00	Reference	0·63	0·33, 1·21	0·18	0·08, 0·40	< 0·001

MET, metabolic equivalent of task.

Low muscle mass was defined based on the definition by the Asian Working Group for Sarcopenia 2019. The recommended cut-offs for appendicular skeletal muscle mass/height^2^ were determined by bioelectrical impedance analysis were < 7·0 kg/m^2^ and < 5·7 kg/m^2^ for men and women, respectively.

Significance level after Bonferroni correction for multiple testing of interaction: *P* for interaction < 0·003.

* Participants were divided into two groups: middle-aged adults (< 60 years) and older adults (≥ 60 years).

† Obesity was defined as body fat percentage ≥ 25 % for men and ≥ 30 % for women.

‡ Participants were divided into two groups using the median leisure-time physical activity by sex: physically inactive adults (< 480 MET-min/week for men and < 240 MET-min/week for women) and physically active adults (≥ 480 MET-min/week for men and ≥ 240 MET-min/week for women).

§ Adjusted for age (years), sex (men, women), cohort (cohort C, cohort D), body fat (%), marital status (married, unmarried), education level (junior high or high school, junior college or technical college, university or higher), household income (< 3 000 000 JPY, 3 000 000–4 999 999 JPY, 5 000 000–6 999 999 JPY, 7 000 000–9 999 999 JPY, ≥ 10 000 000 JPY), cigarette smoking status (current smoker, former smoker, never smoked), alcohol consumption (< 1 d/week, 1–3 d/week, 4–6 d/week, every day), antihypertensive drug usage (user, non-user), anti-dyslipidaemic drug usage (user, non-user), energy intake (kcal/d), protein intake (g/d), green tea consumption (< 1 cup/week, 1–3 cups/week, 4–6 cups/week or 1 cup/d, ≥ 2 cups/d) and leisure-time physical activity (MET-min/week).

|| Adjusted for age (years), cohort (cohort C, cohort D), body fat (%), marital status (married, unmarried), education level (junior high or high school, junior college or technical college, university or higher), household income (< 3 000 000 JPY, 3 000 000–4 999 999 JPY, 5 000 000–6 999 999 JPY, 7 000 000–9 999 999 JPY, ≥ 10 000 000 JPY), cigarette smoking status (current smoker, former smoker, never smoked), alcohol consumption (< 1 d/week, 1–3 d/week, 4–6 d/week, every day), antihypertensive drug usage (user, non-user), anti-dyslipidaemic drug usage (user, non-user), energy intake (kcal/d), protein intake (g/d), green tea consumption (< 1 cup/week, 1–3 cups/week, 4–6 cups/week or 1 cup/d, ≥ 2 cups/d) and leisure-time physical activity (MET-min/week).

¶ Adjusted for age (years), sex (men, women), cohort (cohort C, cohort D), body fat (%), marital status (married, unmarried), education level (junior high or high school, junior college or technical college, university or higher), household income (< 3 000 000 JPY, 3 000 000–4 999 999 JPY, 5 000 000–6 999 999 JPY, 7 000 000–9 999 999 JPY, ≥ 10 000 000 JPY), cigarette smoking status (current smoker, former smoker, never smoked), alcohol consumption (< 1 d/week, 1–3 d/week, 4–6 d/week, every day), anti-dyslipidaemic drug usage (user, non-user), energy intake (kcal/d), protein intake (g/d), green tea consumption (< 1 cup/week, 1–3 cups/week, 4–6 cups/week or 1 cup/d, ≥ 2 cups/d) and leisure-time physical activity (MET-min/week).

## Discussion

In this cross-sectional study on middle-aged and older adults, we investigated the association between coffee consumption and low muscle mass prevalence, focusing on possible effect modification by demographic, health status-related and physical activity-related covariates. We found an inverse association between coffee consumption and the prevalence of low muscle mass. There were no significant interactions among the various covariates after Bonferroni correction.

Epidemiological studies on the association between coffee consumption and muscle mass are scarce; we are only aware of three cross-sectional studies. The Korean National Health and Nutrition Examination Survey in 2008–2011, which included 1781 men aged 60 years and older, reported an inverse association between coffee consumption and low muscle mass prevalence (determined by ASM/height^2^) when measured using dual-energy X-ray absorptiometry^(11)^. They showed that men consuming ≥ 3 cups/d of coffee had a multivariable-adjusted OR (95 % CI) for low muscle mass prevalence of 0·44 (0·21, 0·94) compared to men consuming < 1 cup/d of coffee, after adjusting for age, smoking status, alcohol consumption, exercise, education level, household income, occupation status, protein intake and energy intake. Although the different methods of measurement and analysis preclude simple comparisons, this multivariable-adjusted OR was comparable to that in our analysis of men (OR = 0·42, 95 % CI = 0·19, 0·89). The Japan Multi-Institutional Collaborative Cohort Study in the Saga region, which included 6369 individuals aged 45–74 years, reported that higher coffee consumption was associated with a greater skeletal muscle mass/height^2^ (measured using a multifrequency BIA) in both men and women, after adjusting for age, fat mass index, smoking status, alcohol consumption, green tea consumption, energy intake, protein intake, anti-inflammatory drug usage, antihypertensive drug usage, antihyperlipidaemic drug usage and physical activity level (*β* = 0·023 for men, *β* = 0·011 for women)^(10)^. In addition, the Korean National Health and Nutrition Examination Survey in 2009–2010, which included 6906 men and women aged 40 years and older, examined the association between coffee consumption (< 1 time/d, 1 cup/d, 2 cups/d and ≥ 3 cups/d) and low muscle mass prevalence (determined by ASM/height^2^ measured using dual-energy X-ray absorptiometry). The results showed a lower prevalence of low muscle mass in men consuming 1 cup/d of coffee compared to those consuming coffee < 1 time/d (OR = 0·69, 95 % CI = 0·50, 0·94) but not in women, after adjusting for age, smoking status, alcohol consumption, macronutrients intake, exercise and family income, suggesting that light coffee consumption has a protective effect on the loss of muscle mass in men but not in women^(12)^. The findings of these studies support the results of our study that coffee consumption is associated with a lower prevalence of low muscle mass. To the best of our knowledge, only one longitudinal study has examined the association between coffee consumption and muscle mass. The Danish part of the Monitoring Trends and Determinants in Cardiovascular Disease cohort, which included 2128 men and women (median age of 46 years), did not find a clear association between changes in coffee consumption and changes in fat-free mass/height^2^ measured using a BIA, after adjusting for baseline measures of coffee consumption and fat-free mass/height^2^, age, sex, smoking status, physical activity, education level and menopausal status for women^(13)^. Further longitudinal observational studies and randomised controlled trials are needed to elucidate the causal relationship between coffee consumption and muscle mass.

In this study, women, older age, lower body fat and lower leisure-time physical activity were associated with a higher prevalence of low muscle mass, and these covariates are well-known risk factors of sarcopenia, including low muscle mass^(25)^. The results of this study suggested that there were no interactions between coffee consumption and low muscle mass prevalence with sex and age. Our subgroup analysis by body fat found that the inverse relationship of coffee consumption with low muscle mass prevalence was only noted among adults without obesity, which might be attributed to the fact that adults with obesity rarely had a low muscle mass. In contrast, a meta-analysis of epidemiologic studies reported that higher coffee consumption might be modestly associated with lower adiposity as indicated by BMI and waist circumference^(26)^, which would underestimate the inverse relationship between coffee consumption and low muscle mass prevalence. Our subgroup analyses found that the inverse relationship of coffee consumption with low muscle mass prevalence was only present among physically active adults, but there was no significant interaction after Bonferroni correction. It is well-known that physical activity is one of the most modifiable major lifestyle factors that are essential in maintaining and increasing muscle mass^(1,27,28)^. Coffee is possibly effective in promoting increased muscle mass through a physiological effect on physical activity. A study in rats revealed that low-intensity exercise along with administration of caffeine and lactate compound effectively increased muscle mass, satellite cell activity and anabolic signals^(29)^. However, several studies have reported that caffeine intake might be associated with higher physical activity^(30,31)^. Further research is warranted to elucidate the causal relationships. We also found a tendency for interaction of antihypertensive drugs and coffee consumption with low muscle mass prevalence, but there was no significant interaction after Bonferroni correction. Only 272 of our participants were taking antihypertensive drugs; of these, only 10 had a low muscle mass, resulting in low statistical power. The effect of these factors merits further investigation with larger sample sizes.

Although the molecular mechanism by which coffee affects the maintenance of or increase in skeletal muscle mass is not fully understood, several animal-based studies have been reported (online Supplementary Fig. 2)^(6–8)^. Unlike 4 weeks of normal-water treatment in control mice, 4 weeks of coffee treatment in aged mice increased the skeletal muscle weight, accelerated regeneration of injured muscles and decreased serum pro-inflammatory mediator levels (IL-1*α*, IL-6 and TNF-*α*)^(6)^. Furthermore, coffee increased the proliferation rate and DNA synthesis of satellite cells, which play a central role in the growth and regeneration of skeletal muscle mass, through the Akt signaling pathway^(6)^. In a study using mice, 7 weeks of coffee supplementation suppressed myostatin mRNA expression, increased the expression of an insulin-like growth factor and promoted skeletal muscle hypertrophy^(7)^.

Because sarcopenia, including low muscle mass, can lead to metabolic diseases, falls, fractures and all-cause mortality^(2,3)^, the findings of the present study could contribute to the development of sarcopenia prevention interventions and have a public health impact. Coffee consumption is a modifiable factor, and our findings may facilitate further research on new sarcopenia prevention strategies. However, our study has several limitations. First, because this was a cross-sectional study, causality could not be determined. Second, there is a possibility that residual and unmeasured confounders affected our results. However, the E-value of 6·6 in this study is considered large, indicating that the effects of confounding factors are unlikely to have significantly affected the results. Third, the consumption frequency of coffee was assessed using a single item. The quantity of one cup of coffee was assumed to be a typical one cup (150–173 ml) in the questionnaire. Therefore, information on specific quantities and types of coffee could not be obtained. However, the use of sugar in coffee and tea (always, sometimes and none) was also assessed in the BDHQ and was taken into account in the energy intake of the covariate. Fourth, because leisure-time physical activity was self-reported by the study participants, there is a possibility of overreporting due to social desirability^(32)^. However, overreporting of physical activity generally causes an underestimation of the true effects on the health benefits of physical activity. Although muscle-strengthening exercise is heavily influential on muscle mass, the Global Physical Activity Questionnaire we used to assess leisure-time physical activity could not assess the effect of muscle-strengthening exercises. Further research is needed to examine the effect of different types of leisure-time physical activities on the association between coffee consumption and muscle mass. Finally, the generalisability of the results may be limited because the study participants were Waseda University alumni and their spouses who participated voluntarily, and the participants in cohorts C and D voluntarily selected one of the four courses (cohorts A–D) with different measurement items. Although there were no severe differences in the participant characteristics between cohorts A–B and C–D that would change the direction of our results (data not shown), the results might have been affected by selection bias. Moreover, because only a few participants reported heavy coffee consumption (i.e., ≥ 4 cups/d), we could not adequately examine the effect of heavy coffee consumption. Longitudinal studies involving several populations and randomised controlled trials are required to resolve these issues.

In conclusion, our cross-sectional study revealed that coffee consumption may be inversely associated with the prevalence of low muscle mass. Further longitudinal studies with large representative samples are necessary for a definitive assessment of causality.

## Supplementary material

For supplementary material accompanying this paper visit https://doi.org/10.1017/S0007114522003099. click here to view supplementary material
