# Supplementary material for: Coffee consumption and skeletal muscle mass: WASEDA’S Health Study
Source: Br J Nutr. 2022 Sep 29;130(1):127–36. doi: 10.1017/S0007114522003099 (PMC10244011; doi:10.1017/S0007114522003099)
Supplement: Supplementary file 1 [file S0007114522003099sup001.docx]

Participants who underwent muscle mass measurements
in cohorts C and D between March 2015 and March 2020 (*n* = 2538)

Incomplete survey data (*n* = 101)

- Internet-based questionnaires (*n* = 81)
- Dietary survey (*n* = 20)

Extreme levels of self-reported (*n* = 36)

- Energy intake (*n* = 10)
- Physical activity (*n* = 26)

Pregnant or lactating status (*n* = 14)

History of diseases (*n* = 302)

- Cancer (*n* = 146)
- Stroke (*n* = 28)
- Heart diseases (*n* = 41)
- Kidney failure (*n* = 5)
- Liver cirrhosis and hepatitis (*n* = 9)
- Diabetes mellitus (*n* = 73)

Participants included in the present analysis (*n* = 2085)

**Supplementary Fig. 1.** Flow diagram of the study participants.

**Supplementary Table 1.** Odds ratios for the prevalence of low muscle mass according to coffee consumption (eight groups)

| Coffee consumption | No. of participants | No. of cases | No. of cases per 1000 participants | Model 1 | | Model 2 | |
| --- | --- | --- | --- | --- | --- | --- | --- |
|  |  |  |  | OR | 95% CI | OR | 95% CI |
| none | 120 | 11 | 92 | 1.00 | Reference | 1.00 | Reference |
| <1 cup/week | 88 | 10 | 114 | 1.48 | 0.59, 3.72 | 1.58 | 0.59, 4.24 |
| 1 cup/week | 67 | 8 | 119 | 1.47 | 0.55, 3.94 | 1.80 | 0.64, 5.11 |
| 2–3 cups/week | 161 | 7 | 43 | 0.44 | 0.16, 1.19 | 0.44 | 0.16, 1.23 |
| 4–6 cups/week | 140 | 7 | 50 | 0.56 | 0.21, 1.52 | 0.59 | 0.21, 1.68 |
| 1 cup/day | 539 | 36 | 67 | 0.69 | 0.34, 1.41 | 0.66 | 0.31, 1.42 |
| 2–3 cups/day | 834 | 29 | 35 | 0.37 | 0.18, 0.77 | 0.34 | 0.16, 0.75 |
| ≥4 cups/day | 136 | 5 | 37 | 0.45 | 0.15, 1.35 | 0.39 | 0.12, 1.24 |
| *P* for trend |  |  |  | <0.001 | | <0.001 | |

CI, confidence interval; MET, metabolic equivalent of task; OR, odds ratio.

Low muscle mass was defined based on the definition by the Asian Working Group for Sarcopenia 2019. The recommended cut-offs for appendicular skeletal muscle mass/height^2^ determined by bioelectrical impedance analysis were <7.0 kg/m^2^ and <5.7 kg/m^2^ for men and women, respectively.

Model 1: Adjusted for age (years) and sex (men, women).

Model 2: Adjusted for model 1 plus cohort (cohort C, cohort D), body fat (%), marital status (married, unmarried), education level (junior high or high school, junior college or technical college, university or higher), household income (<3,000,000 JPY, 3,000,000–4,999,999 JPY, 5,000,000–6,999,999 JPY, 7,000,000–9,999,999 JPY, ≥10,000,000 JPY), cigarette smoking status (current smoker, former smoker, never smoked), alcohol consumption (<1 day/week, 1–3 days/week, 4–6 days/week, every day), antihypertensive drug usage (user, non-user), anti-dyslipidaemic drug usage (user, non-user), energy intake (kcal/day), protein intake (g/day), green tea consumption (<1 cup/week, 1–3 cups/week, 4–6 cups/week or 1 cup/day, ≥2 cups/day), and leisure-time physical activity (MET-min/week).


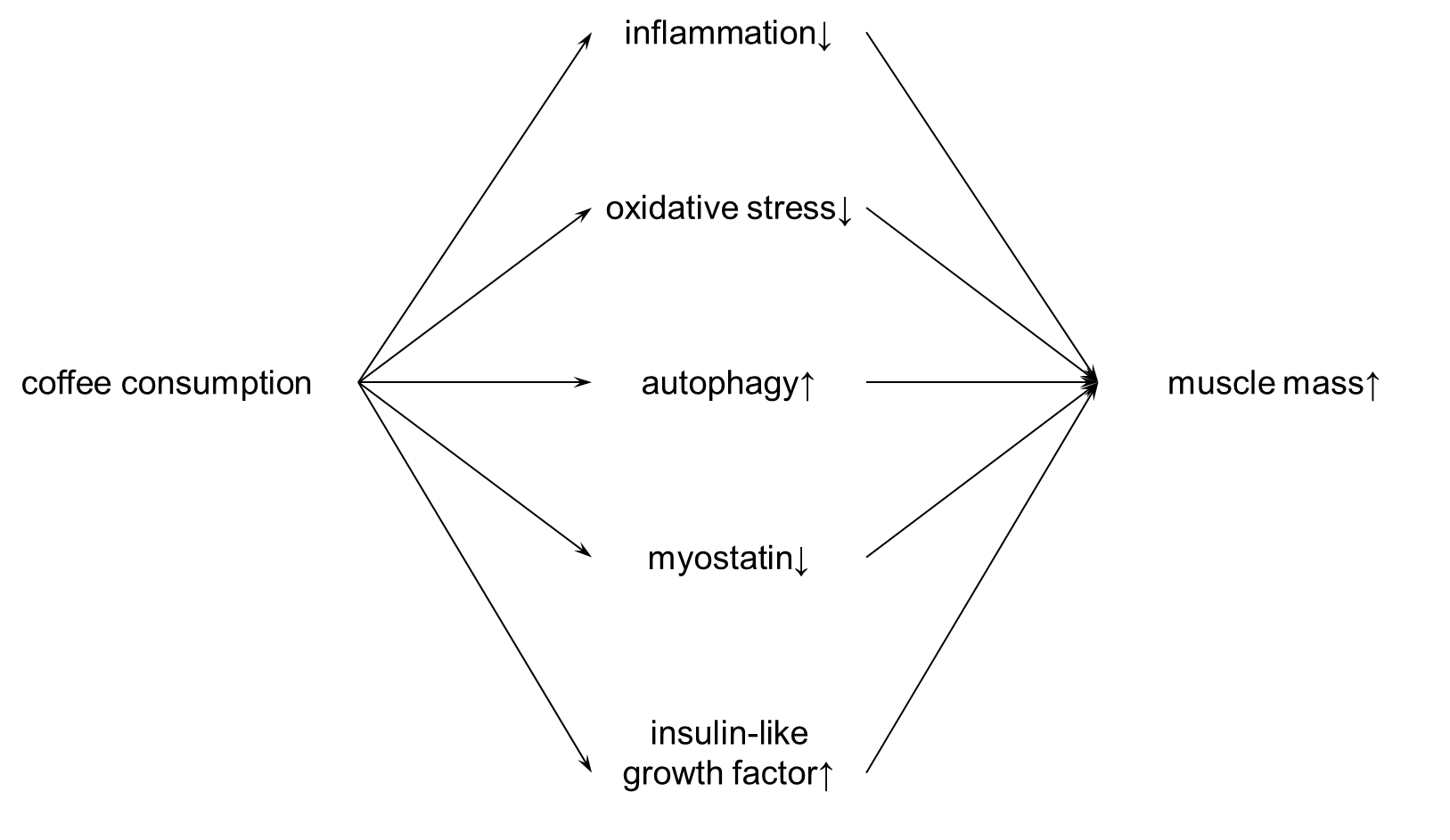


**Supplementary Fig. 2.** Possible physiological effects of coffee consumption on increased muscle mass^(6, 7, 8)^.
